# The CpxA/CpxR two-component system mediates regulation of *Actinobacillus pleuropneumoniae* cold growth

**DOI:** 10.3389/fmicb.2022.1079390

**Published:** 2022-12-23

**Authors:** Qing Yao, Tingting Xie, Yu Fu, Jiajia Wan, Wendie Zhang, Xuejun Gao, Jing Huang, Diangang Sun, Fuxian Zhang, Weicheng Bei, Liancheng Lei, Feng Liu

**Affiliations:** ^1^College of Animal Sciences, Yangtze University, Jingzhou, Hubei, China; ^2^School of Foreign Languages, Zhejiang Gongshang University, Hangzhou, Zhejiang, China; ^3^State Key Laboratory of Agricultural Microbiology, College of Veterinary Medicine, Huazhong Agricultural University, Wuhan, Hubei, China; ^4^College of Veterinary Medicine, Jilin University, Changchun, China

**Keywords:** *Actinobacillus pleuropneumoniae*, two-component system, CpxA/CpxR, cold stress, *cspC*

## Abstract

**Introduction:**

To survive in various hostile environments, two-component system is an adaptive mechanism for diverse bacteria. Activity of the CpxA/CpxR two-component system contributes to coping with different stimuli, such as pH, osmotic and heat stress.

**Methods:**

However, the role of the CpxA/CpxR system in cold resistance is little-known. In this study, we showed that CpxA/CpxRwas critical for *A. pleuropneumoniae* growth under cold stress.

**Results:**

*β*-Galactosidaseanalysis showed that CpxA/CpxR positively regulated the predicted cold stress gene *cspC*. The mutant for cold stress gene *cspC* was impaired in the optimal growth of *A. pleuropneumoniae* under cold stress. Furthermore, electrophoretic mobility shift assays demonstrated that CpxR-P could directly regulate the transcription of the cold stress gene *cspC*.

**Discussion:**

These results presented in this study illustrated that the CpxA/CpxR system plays an important role in cold resistance by upregulating expression of *CspC*. The data give new insights into how *A. pleuropneumoniae* survives in cold stress.

## Introduction

*Actinobacillus pleuropneumoniae* is an important swine pathogen responsible for respiratory infectious disease, porcine contagious pleuropneumonia (PCP). PCP is characterized by fibrinous, hemorrhagic, and necrotic lung lesions, and causes substantial losses in the swine industry worldwide (Guitart-Matas et al., 2022; Zhang et al., 2022). To date, 19 reference serovars have been identified based on the composition of the capsular polysaccharide (CPS; Stringer et al., 2021; Scherrer et al., 2022). Previous studies have reported that five putative two-component systems (TCS) were found in the genome of *A. pleuropneumoniae*, such as CpxR/CpxA, ArcA/ArcB, QseB/QseC, NarP/NarQ, and PhoB/PhoR (Xu et al., 2008).

The CpxR/CpxA system is commonly used by Gram-negative bacteria to regulate many bacterial processes, mainly triggered by a wide range of environmental conditions, such as pH, osmolarity and temperature (Nakayama and Watanabe, 1995; Tschauner et al., 2014; Liu et al., 2022a). The CpxR/CpxA system is composed of the transmembrane sensor kinase CpxA and the cytoplasmic response regulator CpxR (Danese et al., 1995; Raivio and Silhavy, 1997). When bacteria are exposed to extracytoplasmic stresses, the histidine kinase CpxA autophoshorylates and then transfers a phosphoryl group to the response regulator CpxR (Price and Raivio, 2009; Raivio, 2014). Phosphorylation enables CpxR to bind to the promoter region of multiple genes, and alters the transcription of these genes (Raivio et al., 2013).

Bacteria have evolved various complicated networks to cope with different stress factors, such as temperature, pH and mosmotic (Telhig et al., 2020). When subjected to a sudden drop in temperatures, microbes undergo severe unfavorable disturbances such as decreased membrane fluidity and ribosome efficiency, and increase formation of stable secondary structures in nucleic acids (Goto et al., 2015). To adapt to the extreme environments, bacteria activate the expression of cold shock proteins (Csp) that function as general RNA or DNA chaperones to eliminate their secondary structures (Kloska et al., 2020). Csp protein are ubiquitous in a broad variety of bacteria, and multiple variants of this protein have been found, such as CspA, CspB, CspC, CspD, CspE, CspF, CspG, CspH and CspI in *E. coli* (Derzelle et al., 2000).

Porcine pleuropneumonia caused by *A. pleuropneumoniae* leads to economic losses to affected pig farmers. Before causing infection in the host, *A. pleuropneumoniae* must well cope with different environmental cues *in vitro*. However, the cold adaptation mechanism of *A. pleuropneumoniae* is poorly understood. In the present study, we found that the CpxA/CpxR system plays an important role in APP resistance to cold stress. Furthermore, we investigated the mechanism of the Cpx-mediated cold resistance, and showed that the CpxA/CpxR-CspC pathway contribute to the cold stress response in *A. pleuropneumoniae*.

## Materials and methods

### Bacterial strains, culture conditions, and plasmids and primers

The bacterial strains and plasmids, as well as primers used in this study, are listed in Tables 1,2. *A. pleuropneumoniae* cells were grown in tryptic soy broth (TSB; Solarbio, China) or on TSB agar plates supplemented with 10 μg/ml NAD (Sigma-Aldrich, United States) and 10% fetal bovine serum (FBS; Every Green, China). *E. coli* cells were grown in LB medium supplemented with 50 μg/ml diaminopimelic acid (Sigma-Aldrich, United States) or relevant antibiotics (chloramphenicol, 5 μg/ml; kanamycin, 50 μg/ml). The Δ*cspC*, Δ*cspD* and CΔ*cspC* strains were generated using the suicide plasmid pEMOC2 and the shuttle plasmid pJFF224-XN as described earlier (Liu et al., 2022a). In order to induce gene expression, plasmid pJFF224-P*cspC* which expressed the *cspC* gene under the control of IPTG-inducible promoter was constructed, and electrically transferred to the mutant strain Δ*cpxAR*.

**Table 1:** Bacterial strains and plasmids used in this study.

**Strains/plasmids**	**Characteristics**	**Source/reference**
***Actinobacillus pleuropneumoniae***		
S4074	*A. pleuropneumoniae* reference strain of serovar 1; WT strain	From Prof. Weicheng Bei
Δ*cpxAR*	*A. pleuropneumoniae* 4,074 *cpxAR*-deletion mutant	From Prof. Weicheng Bei
Δ*cspC*	*A. pleuropneumoniae* 4,074 *cspC*-deletion mutant	This study
Δ*cspD*	*A. pleuropneumoniae* 4,074 *cspD*-deletion mutant	This study
CΔ*cpxAR*	Complemented strain of Δ*cpxAR*; Cm^r^	From Prof. Weicheng Bei
CΔ*cspC*	Complemented strain of Δ*cspC*; Cm^r^	This study
***Escherichia coli***		
*DH5a*	Cloning host for recombinant vector	Takara
*β2155*	Transconjugation donor for constructing mutant strain	From Prof. Weicheng Bei
***Plasmid***		
pEMOC2	Transconjugation vector: ColE1 ori mob RP4 sacB, Amp^r^Cm^r^	From Prof. Weicheng Bei
pEΔ*cspC*	Up- and down-stream arms of *cspC* were ligated sequentially into pEMOC2, and used as the transconjugation vector for *cspC* gene deletion	This study
pEΔ*cspD*	Up- and down-stream arms of *cspD* were ligated sequentially into pEMOC2, and used as the transconjugation vector for *cspD* gene deletion	This study
pJFF224-XN	E. coli-APP shuttle vector: RSF1010 replicon; mob oriV, Cm^r^	From Prof. Weicheng Bei
pCΔ*cspC*	pJFF224-XN carrying the intact *cspC*	This study
pET-30a	Expression vector; Kan^r^	Novagen
pET30a-*cpxR*	pET-30a carrying *cpxR* gene	Our Laboratory

Cm^r^, Chloramphenicol resistance; Amp^r^, Ampicillin resistance; Kan^r^, Kanamycin resistance.

**Table 2:** Primers used in this study.

Primer	Sequence (5′–3′) a	Use
*cspC*-S-F	CTGTCGACAAGACGGGATGGCGGAAGAT	amplification of *cspC* upstream homology arms
*cspC*-S-R	CAACAGGTATCGTTAAATGGTTCAACTTCAGCGGTAAACGTAAAAACACT	amplification of *cspC* upstream homology arms
*cspC*-X-F	AGTGTTTTTACGTTTACCGCTGAAGTTGAACCATTTAACGATACCTGTTG	amplification of *cspC* downstream homology arms
*cspC*-X-R	ATGCGGCCGCTCTTTGCGTTGCTCTTTTCGCTGTT	amplification of *cspC* downstream homology arms
*cspC*-W-F	TTGTATCCGCTGGCGTATGA	detection exterior of *cspC* mutants
*cspC*-W-R	TAGCGTTCTTGAGTACGATTGCTT	detection exterior of *cspC* mutants
*cspC*-N-F	GGACCGCGCTCTGAATCTTGAACTT	detection interior of *cspC* mutants
*cspC*-N-R	GGAAAATCCTATGTCTAAAGCAACA	detection interior of *cspC* mutants
*cspC*-C-F	CCGCTCGAGAAGTCCAAATAGATGCCGACCCAAT	amplification of *cspC*
*cspC*-C-R	AAGGAAAAAAGCGGCCGCTTAAAGTGTTTTTACGTTTACCGCT	amplification of *cspC*
*cspD*-S-F	ACGCGTCGACAGACAAAGTCGGTATCCCAGG	amplification of *cspD* upstream homology arms
*cspD*-S-R	GATGCACCGCGTTCACCGTTAAACCATTTCACGATGCCAACTTCCA	amplification of *cspD* upstream homology arms
*cspD*-X-F	TGGAAGTTGGCATCGTGAAATGGTTTAACGGTGAACGCGGTGCATC	amplification of *cspD* downstream homology arms
*cspD*-X-R	AAGGAAAAAAGCGGCCGCAGCGGCGGTAACAATAGAACT	amplification of *cspD* downstream homology arms
*cspD*-W-F	CCGCCACTCCAGCAAAATACC	detection exterior of *cspD* mutants
*cspD*-W-R	TGTACCTTTTGACCTACTTTAAGCG	detection exterior of *cspD* mutants
*cspD*-N-F	CAATAGTGCGAAAGGATTCGGATTT	detection interior of *cspD* mutants
*cspD*-N-R	TGTACCTTTTGACCTACTTTAAGCG	detection interior of *cspD* mutants
CpxR-F	CGCCCATATGATGCCTAGAATTTTACTCGTTG	amplification of *cpxR*
CpxR-R	CGCCCTCGAGTTTTTCAGTAACGAGTAAATAACCTCGACCGC	amplification of *cpxR*
16SrRNA-F	CCATGCCGCGTGAATGA	detection the transcription of 16SrRNA
16SrRNA-R	TTCCTCGCTACCGAAAGAACTT	detection the transcription of 16SrRNA
*cspC*-EMSA-F	TTGATGAAAAGAATTGCTGCACGTT	amplification of *cspC* promoter region for EMSA
*cspC*-EMSA-R	ATTTAAGTCCAAATAGATGCCGACC	amplification of *cspC* promoter region for EMSA
*cspD*-EMSA-F	AACCTCCGTATTTTAAAAAATTTTG	amplification of *cspD* promoter region for EMSA
*cspD*-EMSA-R	CCGAATCCTTTCGCACTATTGAACC	amplification of *cspD* promoter region for EMSA
*rpoE*-EMSA-F	TAAAAAGATAAGATAAGCGGTC	amplification of *rpoE* promoter region for EMSA
*rpoE*-EMSA-R	AGTGTGTAACAAAAATGAAAAGT	amplification of *rpoE* promoter region for EMSA
*rpoD*-EMSA-F	GCGGAAGAAAAGCAAGAGTTGGTCA	amplification of *rpoD* promoter region for EMSA
*rpoD*-EMSA-R	TCCATAATTGTATCCGTTTTGTGTG	amplification of *rpoD* promoter region for EMSA

### RNA extraction, transcriptome analysis and qRT-PCR

Bacterial cells grown overnight in TSB medium at 37°C were subcultured 1:1000 in fresh TSB broth. Cells grown up to the exponential phase (OD600; 0.6) were exposed to 4°C for a series of times. Total RNA was extracted from bacterial cultures using the Bacteria Total RNA Isolation Kit (Sangon Biotech, China). The synthesis of complementary DNA (cDNA) was achieved using the HiScript II Q RT SuperMix for qRT-PCR (Vazyme, China). For quantitative RT-PCR, the reactions were performed using SYBR qPCR Mix (Vazyme, China) and run in the CFX96 Real-Time System (Bio-Red, United States). Data were normalized using the 16S rRNA as internal control, and calculated using the 2^−ΔΔCt^ method.

### *β*-Galactosidase assay

A *cspC/cspD*-lacZ fusions containing the promoter region of *cspC/cspD* and the *lacZ* gene, and cloned into the Xho I and Not I sites of the plasmid pJFF224-XN. Then, the recombinant plasmid was introduced into the wild-type and the ∆*cpxAR* mutant strain. All strains were grown overnight in TSB broth at 37°C, diluted 1:1,000 in fresh TSB broth, and grown at 20°C for a series of times. The collected culture was assayed for *β*-Galactosidase activity by using a *β*-galactosidase (*β*-GAL) Activity Assay Kit (Micromethod; Sangon Biotech) according to the manufacturer’s specification.

### Electrophoretic mobility shift assay

The His6-CpxR protein was expressed using *E. coli* BL21 (DE3)-containing pET30a-CpxR and purified by using a Ni-nitrilotriacetic acid (Ni-NTA) resin affinity chromatography (Qiagen, Germany). The purified protein was phosphorylated by acetyl phosphate (Sigma, United States) according to previously described procedures (Li et al., 2018). DNA probes were amplified and purified and then labeled using a Biotin Labeling Kit (Beyotime, China). Then, the phosphorylated CpxR and labeled probes were used for protein-DNA EMSAs as described previously (Cheng et al., 2021). Labeled DNA probe (1 μM) and various concentrations of phosphorylated CpxR protein (0–4 pmol) were incubated at 24°C for 20 min in reaction buffer (50 mM Tris–HCl, pH 8.0, 2.5 mM MgCl_2_, 100 mM KCl, 0.2 mM DTT, 10% glycerol, 2 μg salmon sperm DNA). For competition experiments with unlabeled DNA probes, a 100-fold molar excess was preincubated with phosphorylated CpxR protein. The reaction mixtures were loaded on a 4% non-denaturing polyacrylamide electrophoresis in 0.5 × Tris-borate-EDTA (TBE) buffer. The bands of labeled probes were subsequently transferred to nylon membrane (Beyotime, China), and detected using the Chemiluminescent EMSA Kit (Beyotime, China).

### Growth assays under cold stress

To measure cold growth, the *A. pleuropneumoniae* strain S4074 and its mutant derivatives were cultured in TSB medium overnight at 37°C, then diluted 1:100 (~1 × 10^7^ CFU/ml) into fresh TSB broth and incubated at 20°C for 48 h. Every 4 h for 48 h, samples were serially diluted and plated on TSB agar, and the OD600 was measured.

### Bioinformatic and statistical analysis

The −10 and − 35 promoter regions, and transcriptional atart site (TSS) of the cold shock gene *cspC* were, respectively, predicated by BPROM (Prediction of bacterial promoters)^1^ and Berkeley Drosophila Genome Project (BDGP).^2^ All data were analyzed using two-tailed Student’s *t*-tests (GraphPad Prism version 7.0, GraphPad Software, inc., San Diego, United States), and presented as mean ± standard deviation (SD). *p* < 0.05 was considered statistically significant.

## Results

### Cpxa/CpxR is required for cold growth in *Actinobacillus pleuropneumoniae*

**Figure 1:**
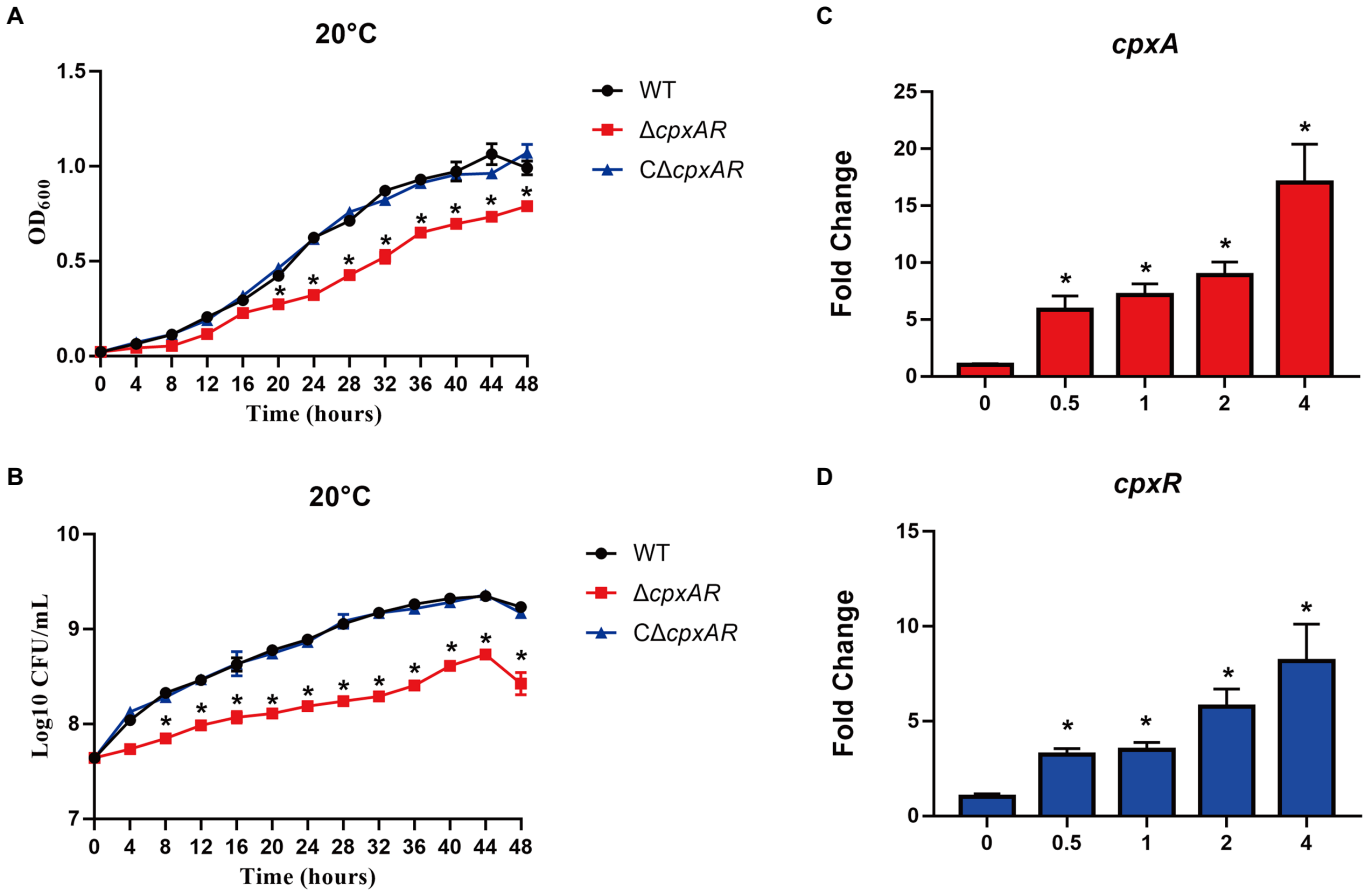
CpxA/CpxR is required for APP cold growth. The growth rates of the WT, ∆*cpxAR* and C∆*cpxAR* strains at 20°C were monitored by measurement of OD600 **(A)** and viable cell counts **(B)**. qRT-PCR analysis of *cpxA* **(C)** and *cpxR* **(D)** genes in *A. pleuropneumoniae* S4074 under 20°C. *p < 0.05.

To investigate whether CpxAR is involved in the cold adaptation of *A. pleuropneumoniae*, we tested the growth rates of the WT, ∆*cpxAR* and C∆*cpxAR* strains when exposed to cold stress (20°C). Under cold stress, the growth rate of the mutant strain ∆*cpxAR* was significantly lower than that of the WT and C∆*cpxAR* strains (Figures 1A,B). Our previous study found that the growth rate of ∆*cpxAR* was also lower than that of the WT at 37°C, and the difference was obviously much smaller than that at 20°C (Li et al., 2018). The qRT-PCR analysis showed that the mRNA level of the *cpxA* and *cpxR* genes were upregulated under cold stress in the WT strain (Figures 1C,D). These results suggest that CpxA/CpxR plays an important role in cold adaptation of *A. pleuropneumoniae*.

### Cpxa/CpxR transcriptionally regulates the expression of *cspC* under cold stress

**Figure 2:**
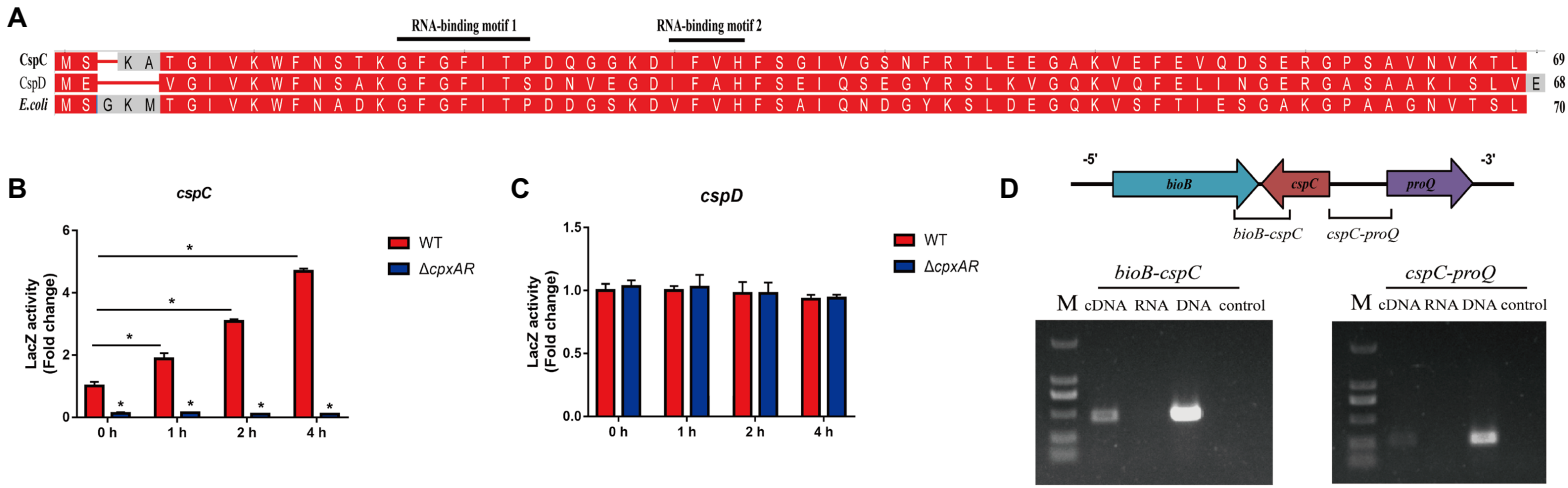
*cspC* transcription is activated by CpxR. **(A)** Alignment of cold shock proteins homologs showing the conserved cold shock domain (CSD), which harbors two nucleic acid-binding motifs RNAP1 and RNAP2. The *A. pleuropneumoniae* CspC and CspD, and *E. coli* CspA are included for comparison. Promoter activity of *cspC* **(B)** and *cspD* **(C)** genes in the WT, ∆*cpxAR* and C∆*cpxAR* strains under 20°C. *p < 0.05. **(D)** Transcriptional characteristics of *cspC* gene as determined by RT-PCR at 20°C.

Analysis of the *A. pleuropneumoniae* genome revealed that it contains two Csp proteins (CspC and CspD) which have a high amino acid identity with *E.coli* CspA (Figure 2A). Each protein possesses a conserved cold shock domain (CSD) that harbors two nucleic acid-binding motifs RNAP1 and RNAP2.

To elucidate the mechanism of CpxA/CpxR affecting APP cold adaptability, *β*-Galactosidase assay was performed to examine the link between CpxR and the cold shock genes *cspC* and *cspD*. As shown in Figures 2B,C, the promoter activity of *cspC*-lacZ under cold stress were significantly elevated in the WT strain, and decreased in the ∆*cpxAR* mutant compared with the former, but the the *cspD* gene was not. These findings indicated that CpxA/CpxR regulates the expression of the cold shock gene *cspC* which is cold inducible in *A. pleuropneumoniae*.

The cold shock gene *cspC* is adjacent to the gene *bioB* and *proQ* in the *A. pleuropneumoniae* chromosome (Figure 2D). In order to describe the *cspC* gene, we performed RT-PCR across the *bioB*-*cspC* and *cspC*-*proQ* junctions. The RT-PCR analysis indicated that the *cspC* gene is transcribed separately from the *proQ* gene.

### Cspc is essential for cold growth in *Actinobacillus pleuropneumoniae*

**Figure 3:**
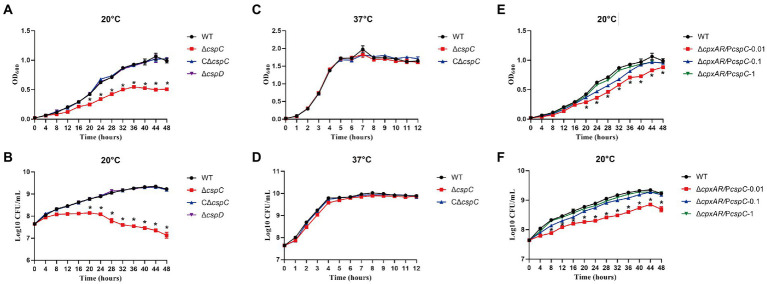
CspC is critical for APP cold growth. The growth rates of the WT, ∆*cspC*, C∆*cspC* and ∆*cspD* strains at 20°C were monitored by measurement of OD600 **(A)** and viable cell counts **(B)**. The growth rates of the WT, ∆*cspC* and C∆*cspC* strains at 37°C were monitored by measurement of OD600 **(C)** and viable cell counts **(D)**. The growth rates of strains expressing *cspC* with IPTG at indicated concentrations were monitored by measurement of OD600 **(E)** and viable cell counts **(F)**. *p < 0.05.

To investigate whether CspC and CspD are involved in cold stress, we generated the ∆*cspC* and ∆*cspD* mutant strains, and the C∆*cspC* complemented strain, and tested the growth rates of the WT, ∆*cspC*, ∆*cspD* and C∆*cspC* strains at 20°C and 37°C. Under cold stress (20°C), the ∆*cspC* mutant exhibited growth defects, whereas the WT, ∆*cspD* and C∆*cspC* strains displayed normal growth (Figures 3A,B). When the bacteria were cultured at 37°C, the growth property of ∆*cspC* mutant was similar to those of the WT and C∆*cspC* strains (Figures 3C,D). The growth of ∆*cpxAR*/P*cspC* in liquid media was then assayed. Clearly, when CspC was produced with IPTG at 0.1 mM and above, the growth defect of the ∆*cpxAR* mutant strain was significantly rescued (Figures 3E,F). These results suggested that CspC is essential for cold growth in *A. pleuropneumoniae*.

### Cpxr binds specifically to the *cspC* promoter region

**Figure 4:**
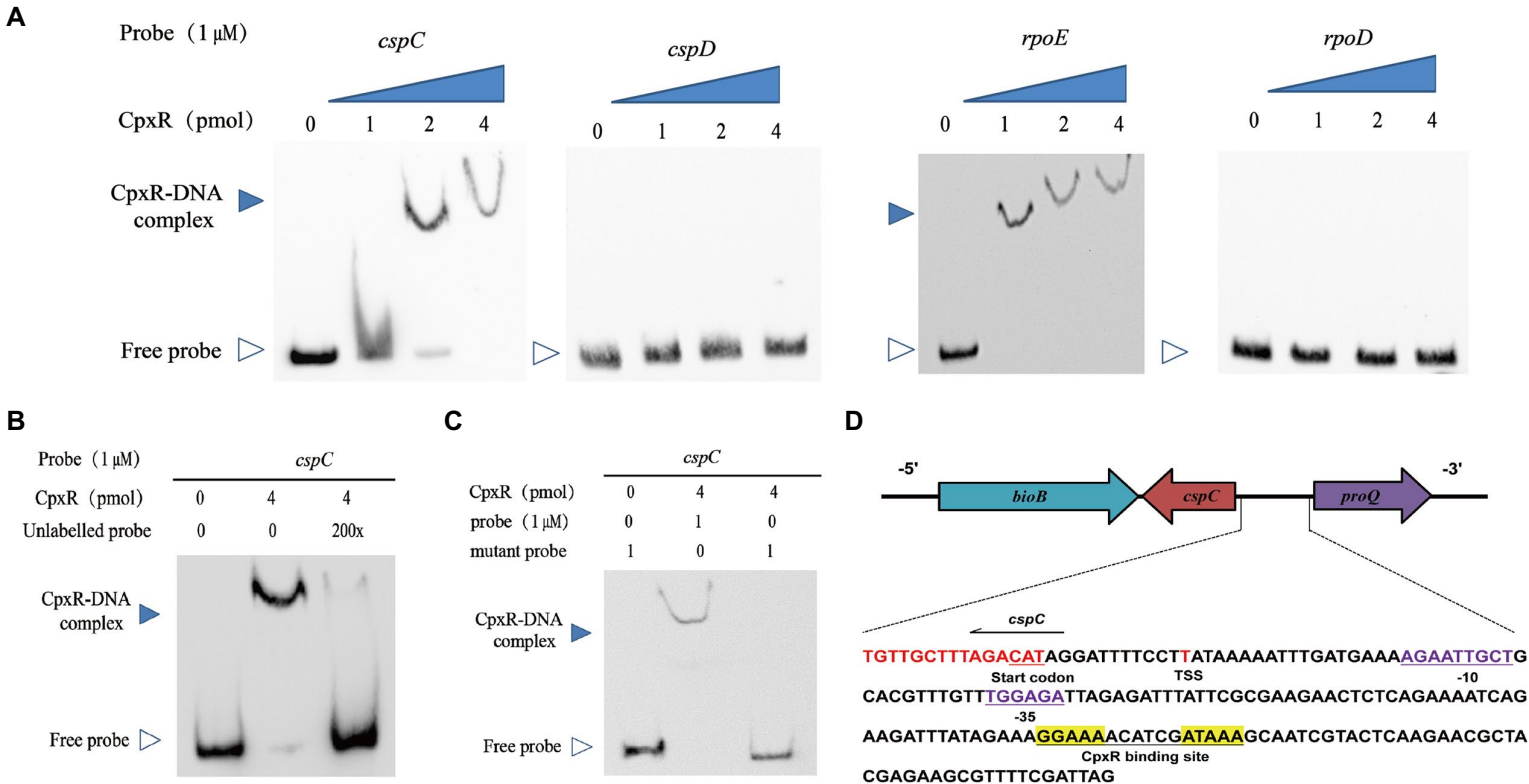
CpxR binds promoter of *cspC* but not that of *cspD*. **(A)** EMSAs analysis of the binding affinity of various amounts of CpxR-P with the *cspC*, *cspD*, *rpoE* (positive control), *rpoD* (negative control) promoter regions. **(B)** EMSAs analysis of the binding of CpxR-P with the *cspC* promoter region under adding excess amounts of unlabeled probes. **(C)** Labeled mutant *cspC* promoter region were incubated with various concentrations of CpxR-P. **(D)** Characterizing the CpxR binding site. The putative CpxR binding site is underlined and shown in yellow background. The −35 and − 10 box are underlined and shown in purple nucleotides. The transcription start site (TSS) is shown in red.

Since CpxR is a response regulator, we examined the interaction between the recombinant CpxR protein and the putative *cspC* promoter region using EMSA. As shown in Figure 4A, incubation of biotin-labeled *cspC* with CpxR protein led to the formation of DNA-protein complexes in a protein concentration-dependent manner. Meanwhile, no CpxR-DNA complex was observed with adding excess amounts of unlabeled probes, suggesting that the CpxR could bind specifically to the *cspC* promoter region (Figure 4B).

Previous studies found that the CpxR binding site has a conserved sequence GTAAA-(N)_4–8_-GTAAA (Bruna et al., 2018; Jia et al., 2022). In this study, we found that a 16-nt region (5’-GGAAAACATCGATAAA-3′) was relatively consistent with the characteristics of conserved sequence. As shown in Figure 4C, CpxR-P was unable to bind to a mutant *cspC* putative promoter region in which the 16-nt region was deleted from the putative CpxR-P binding box. In order to describe the CpxR binding site in detail, the *cspC* promoter region and transcription start site were, respectively, analyzed by BPROM and BDGP. As shown in Figure 4D, a putative −10 AGAATTGC box, −35 TGGAGA box and TSS were detected, and, respectively, located 30 bp, 50 bp and 12 bp upstream of the start codon.

## Discussion

Two-component system (TCS) is a key signal transduction mechanism that controls many aspects of bacterial physiology, sense a broad range of stimuli and make an appropriate response to adapt and survive in changing environmental conditions. The CpxA/CpxR system identified as a pleiotropic TCS in many Gram-negative bacteria, regulates many virulence factors, such as lipopolysaccharide, polysaccharide capsule, and type IV pilus and responds to a variety of extracellular stimuli including salt, heat, metals, and pH (Jubelin et al., 2005; Hunke et al., 2012; Yan et al., 2020; Liu et al., 2022a,b). However, the link between CpxA/CpxR and cold stress is still unknown. In this study, in-frame mutation of the *cpxA* and *cpxR* genes exhibited growth defects, and the mRNA level of the *cpxA* and *cpxR* genes elevated under cold stress, suggesting that CpxA/CpxR plays a crucial role in cold adaptation of *A. pleuropneumoniae*. Furthermore, we provided the first insights into how CpxA/CpxR contributes to cold growth in *A. pleuropneumoniae*.

Cold is an adverse environment for bacteria, altering secondary structures of nucleic acids that render cells nonviable (Balhesteros et al., 2010). To stabilize secondary structures of the RNA, bacteria activate the expression of multiple variants of Csp proteins under cold stress, such as the nine Csp proteins (CspA to CspI) in *E. coli* (Graumann and Marahiel, 1999; Li et al., 2021). In the present study, we found that the genome of *A. pleuropneumoniae* encodes two Csp proteins (CspC and CspD), which are constituted of a single cold shock domain (CSD). Here, *β*-Galactosidase analysis showed that CspC is cold inducible, and positively regulated by CpxA/CpxR. In addition, these cold growth tests revealed that CspC was vital for APP cold adaptability. These results indicated that CspC is involved in the mechanism of CpxA/CpxR-mediated cold stress.

To response to the adverse condition, activated CpxR binds to the promoter region of target genes, and regulates these genes expression. Previous studies showed that the CpxR binding consensus sequence is identified as GTAAA-(N)_4-8_-GTAAA, or TTTAC-(N)_4-8_-TTTAC in many other bacteria (De Wulf et al., 2002; Srinivasan et al., 2012; Feldheim et al., 2016). According to the consensus sequence, we found a potential CpxR binding site (GGAAA-N_6_-ATAAA) located 52 bp upstream of the promoter −35 region, and 95 bp upstream of the transcription start site. Previous studies showed that the CpxR-binding site is generally located upstream of the promoter region and within 100 bp upstream of the transcription start site, primarily functions as a class I factor and activates their transcription (De Wulf and Lin, 2000; Raffa and Raivio, 2002; Yamamoto and Ishihama, 2006). In addition, we verified that CpxR could directly bind to the promoter region of the *cspC* gene, and the CpxR-*cspC* interaction was specific by EMSA. These results revealed that CpxR positively regulates the *cspC* gene transcription.

**Figure 5:**
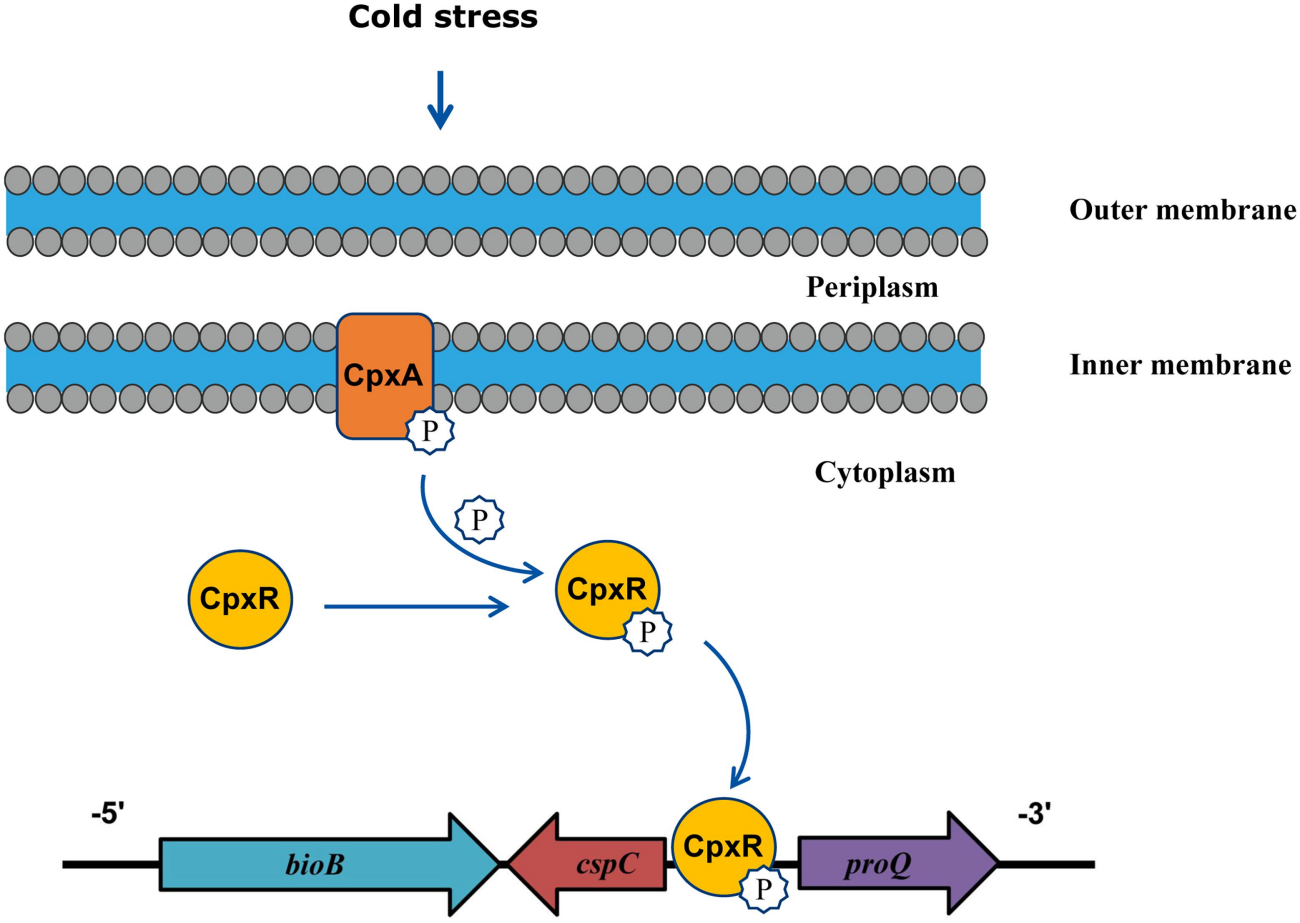
Model illustrating that CpxA/CpxR mediates cold stress in *A. pleuropneumoniae*. Under cold stress, phosphorylated form of CpxA transfers the phosphate group to CpxR, then the active CpxR binds the promoter of the *cspC* gene to upregulate its transcription.

In conclusion, this study illuminates the mechanism of *A. pleuropneumoniae* cold stress and demonstrate the involvement of CpxA/CpxR for the first time. These findings indicated that CpxA/CpxR contributes to cold growth by positively regulating the expression of *cspC* gene (Figure 5). Since the CpxA/CpxR and CspC are conserved in many bacteria, the revelation of the CpxA/CpxR-CspC pathway will provide important implications for elucidating cold stress in other bacteria.

## Data availability statement

The datasets presented in this study can be found in online repositories. The names of the repository/repositories and accession number(s) can be found in the article/Supplementary material.

## Author contributions

FL and LL conceived and designed the experiments. QY, YF, JW, WZ and TX performed the experiments. FL and QY analyzed the data. XG,WB, DS, and FZ contributed reagents, materials, and analysis tools. JH polished the language. All authors contributed to the article and approved the submitted version.

## Funding

This research was supported by the National Natural Science Foundation of China (32002252).

## Conflict of interest

The authors declare that the research was conducted in the absence of any commercial or financial relationships that could be construed as a potential conflict of interest.

## Publisher’s note

All claims expressed in this article are solely those of the authors and do not necessarily represent those of their affiliated organizations, or those of the publisher, the editors and the reviewers. Any product that may be evaluated in this article, or claim that may be made by its manufacturer, is not guaranteed or endorsed by the publisher.

## Footnotes

^1^ http://linux1.softberry.com/berry.phtml?topic=bprom&group=programs&subgroup=gfindb

^2^ https://www.fruitfly.org/seq_tools/promoter.html
