# Supplementary material for: The CpxA/CpxR two-component system mediates regulation of Actinobacillus pleuropneumoniae cold growth
Source: Front Microbiol. 2022 Dec 23;13:1079390. doi: 10.3389/fmicb.2022.1079390 (PMC9816388; doi:10.3389/fmicb.2022.1079390)
Supplement: Supplementary file 1 [file Data_Sheet_1.ZIP › Original data/original data-figure-3/figure-3-C/figure-3-C.pptx]

## Slide 1
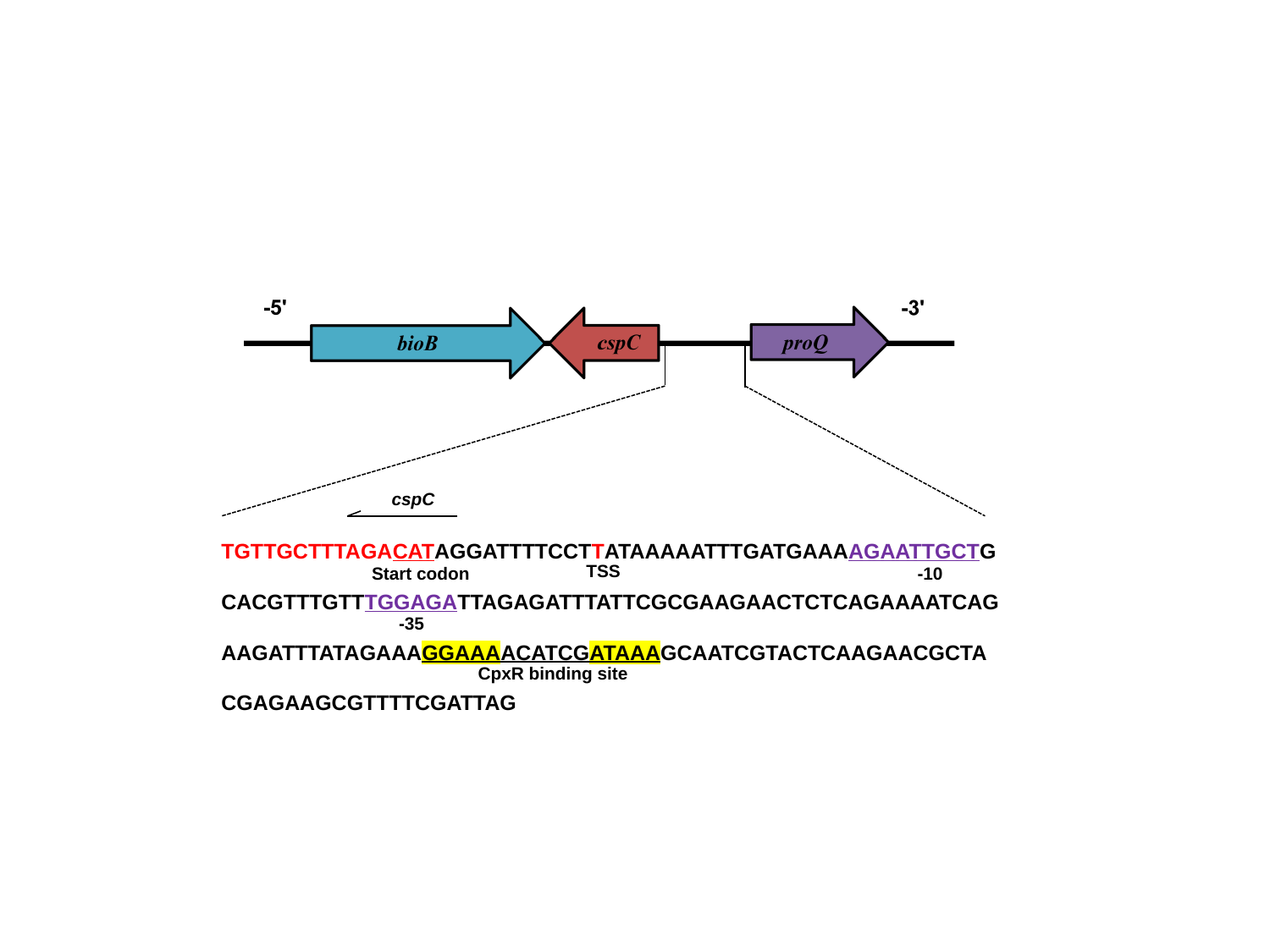

cspC
TGTTGCTTTAGACATAGGATTTTCCTTATAAAAATTTGATGAAAAGAATTGCTGCACGTTTGTTTGGAGATTAGAGATTTATTCGCGAAGAACTCTCAGAAAATCAGAAGATTTATAGAAAGGAAAACATCGATAAAGCAATCGTACTCAAGAACGCTACGAGAAGCGTTTTCGATTAG
TSS
-10
Start codon
-35
CpxR binding site
