# Supplementary figures and images for: The CpxA/CpxR two-component system mediates regulation of Actinobacillus pleuropneumoniae cold growth
Source: Front Microbiol. 2022 Dec 23;13:1079390. doi: 10.3389/fmicb.2022.1079390 (PMC9816388; doi:10.3389/fmicb.2022.1079390)

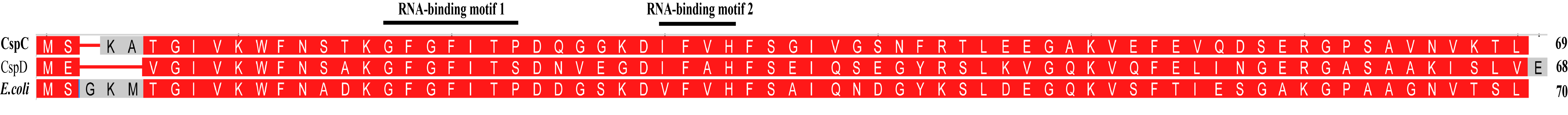

Supplement: Supplementary file 1 [file Data_Sheet_1.ZIP › Original data/original data-figure-2/Figure-1-A/figure-2-A.tif]

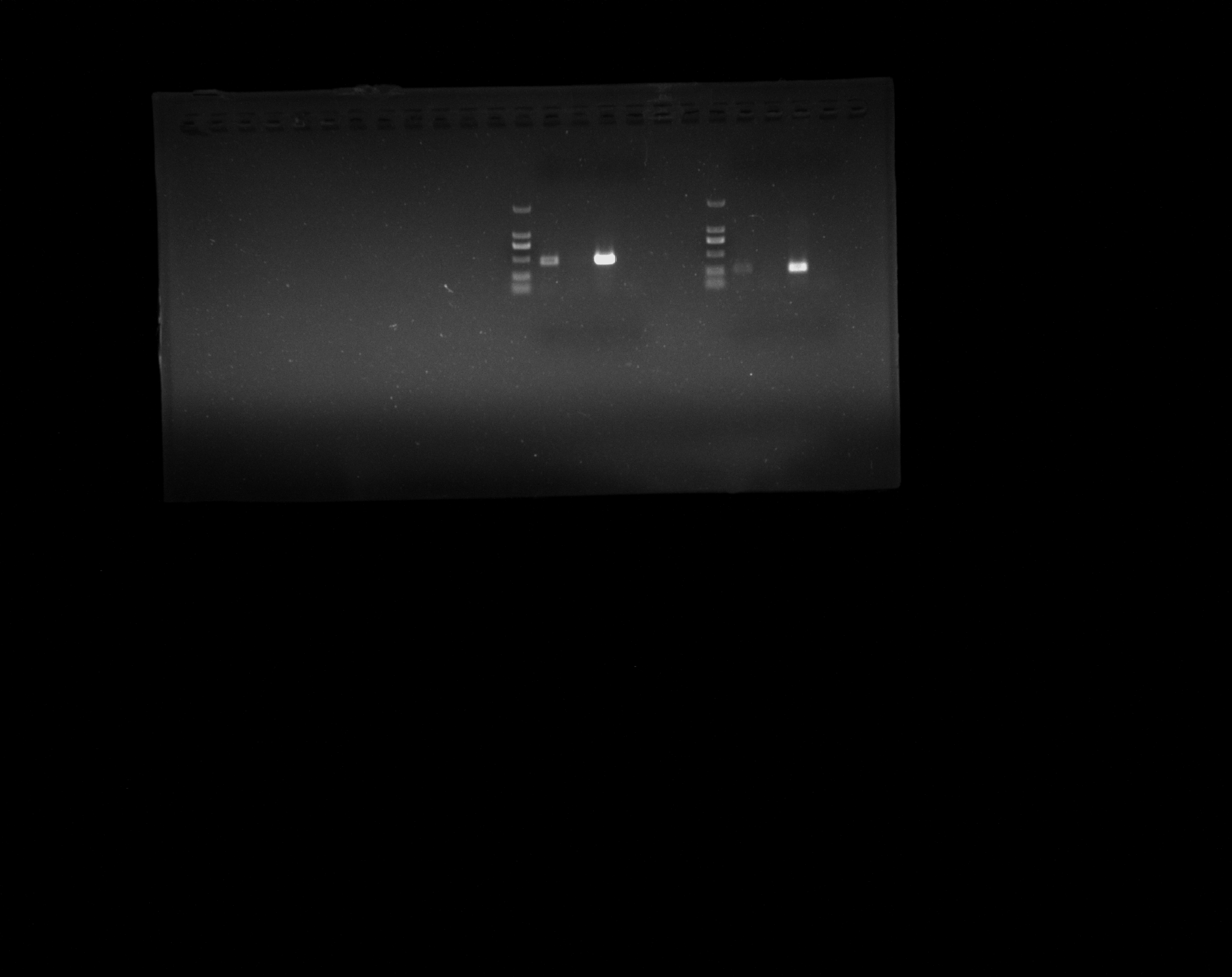

Supplement: Supplementary file 1 [file Data_Sheet_1.ZIP › Original data/original data-figure-2/Figure-1-D/figure-2-D.bmp]

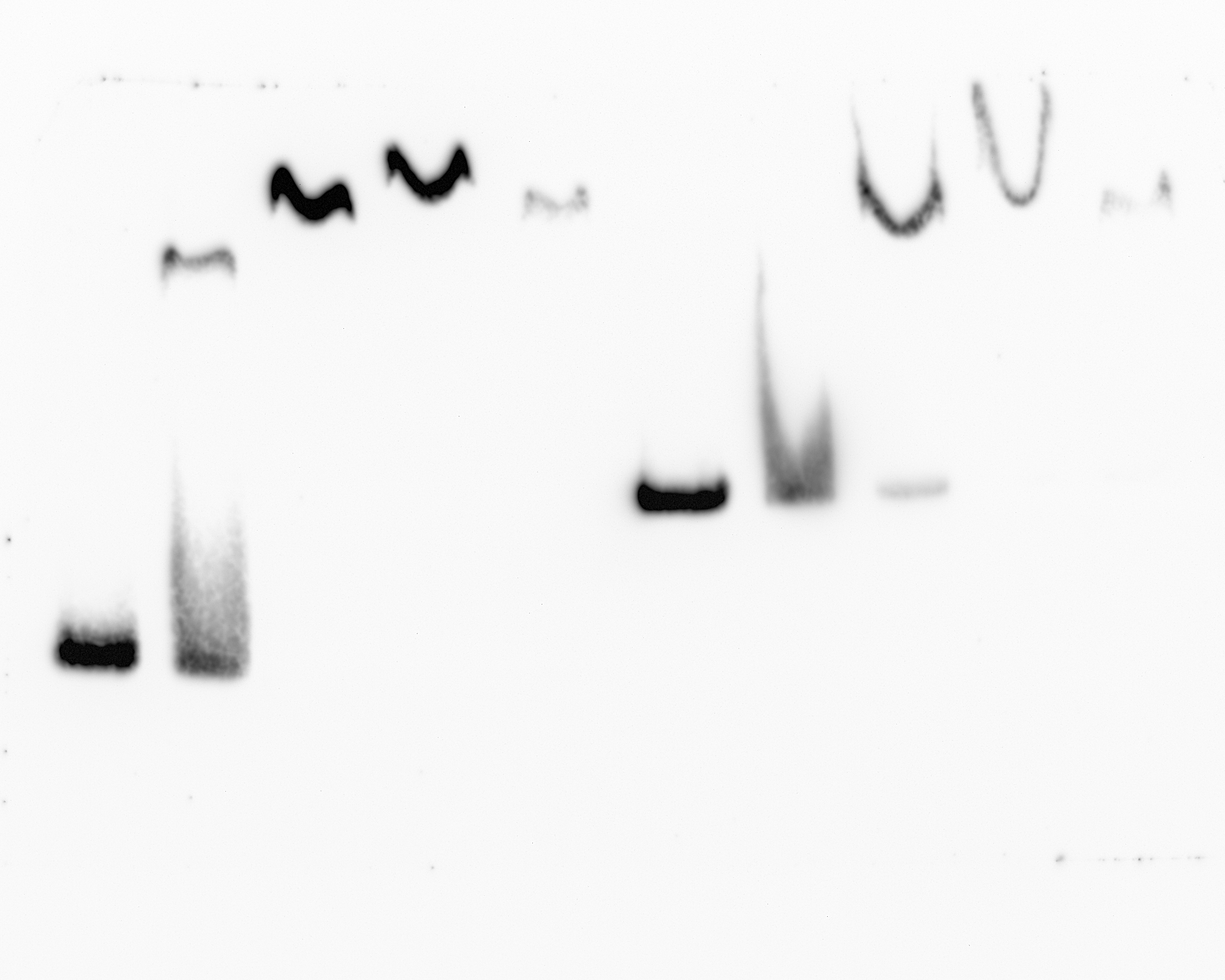

Supplement: Supplementary file 1 [file Data_Sheet_1.ZIP › Original data/original data-figure-3/figure-3A/EMSA-cspC.tif]

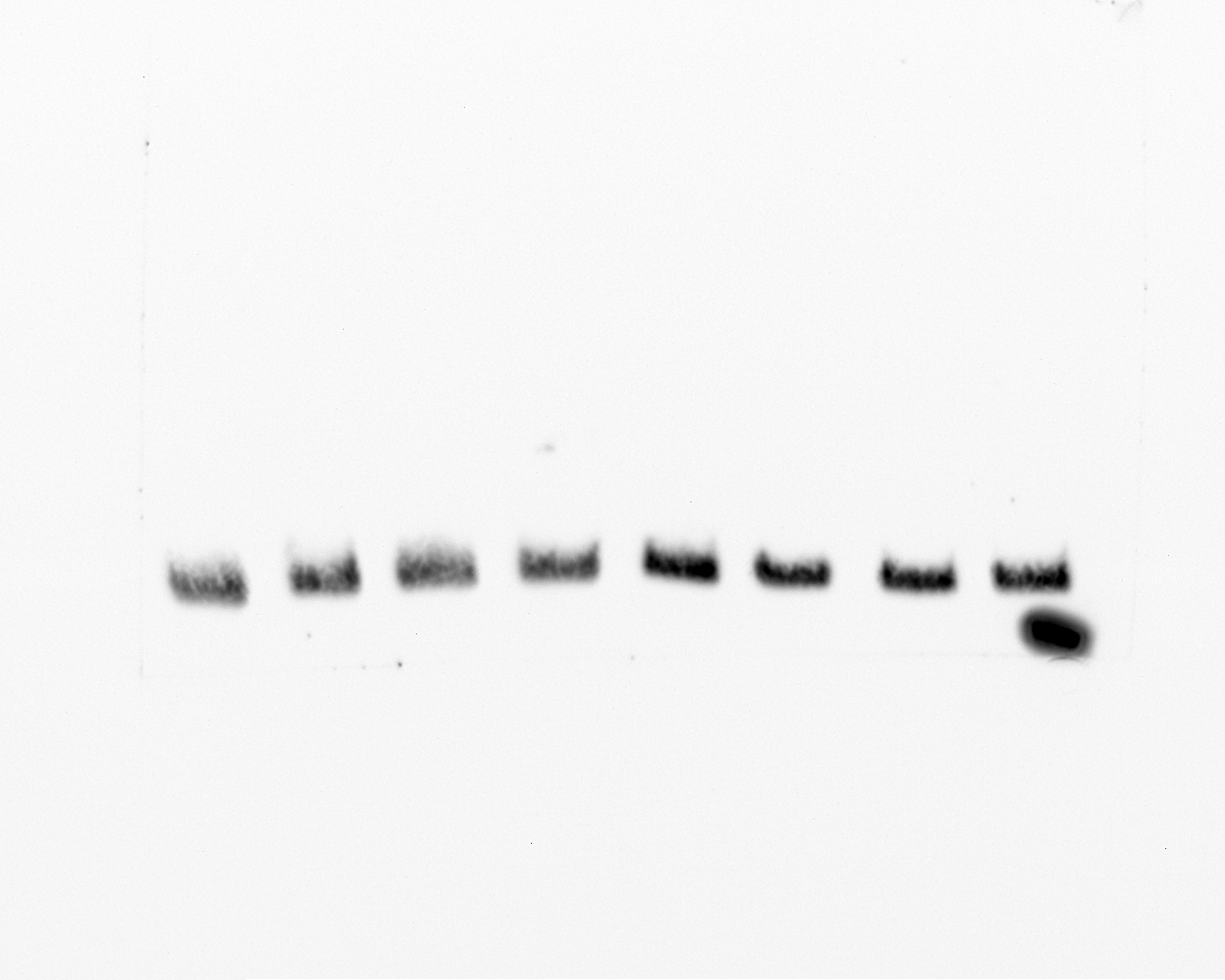

Supplement: Supplementary file 1 [file Data_Sheet_1.ZIP › Original data/original data-figure-3/figure-3A/EMSA-rpoD cspD.tif]

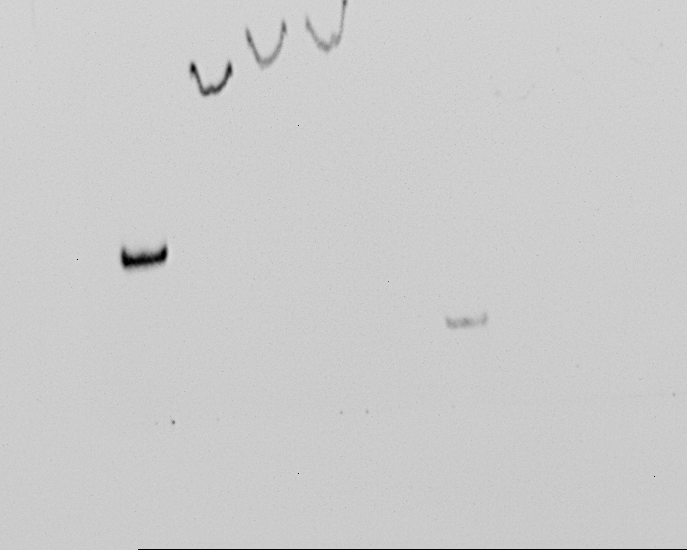

Supplement: Supplementary file 1 [file Data_Sheet_1.ZIP › Original data/original data-figure-3/figure-3A/EMSA-rpoE.tif]

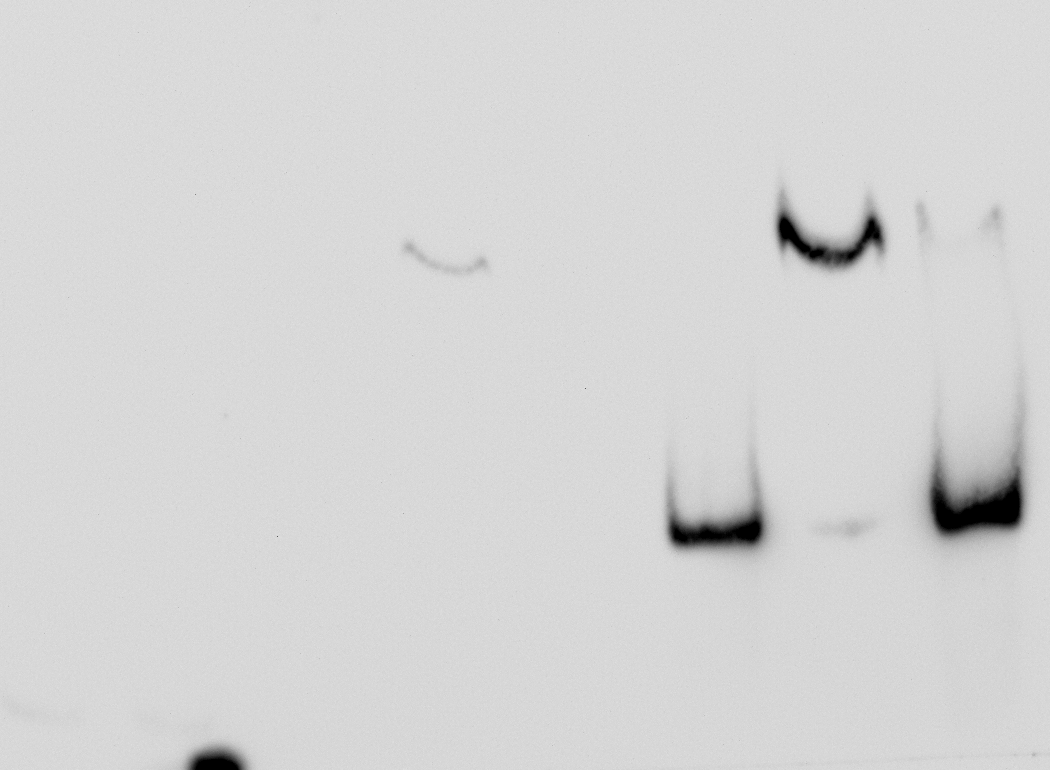

Supplement: Supplementary file 1 [file Data_Sheet_1.ZIP › Original data/original data-figure-3/figure-3B/figure-3-B.tif]
